# Evaluation of Cough Medication Use Patterns in Ambulatory Care Settings in the United States: 2003–2018

**DOI:** 10.3390/jcm11133671

**Published:** 2022-06-25

**Authors:** Seonkyeong Yang, Juan M. Hincapie-Castillo, Xuehua Ke, Jonathan Schelfhout, Helen Ding, Mandel R. Sher, Lili Zhou, Ching-Yuan Chang, Debbie L. Wilson, Wei-Hsuan Lo-Ciganic

**Affiliations:** 1 Department of Pharmaceutical Outcomes & Policy, College of Pharmacy, University of Florida, Gainesville, FL 32611, USA; yang.se@ufl.edu (S.Y.); c.chang@ufl.edu (C.-Y.C.); debbie.wilson@ufl.edu (D.L.W.); 2 Department of Epidemiology, Gillings School of Global Public Health, University of North Carolina at Chapel Hill, Chapel Hill, NC 27599, USA; jhincapie-castillo@unc.edu; 3 Center for Observational and Real-World Evidence (CORE), Merck & Co., Inc., Rahway, NJ 07065, USA; xuehua.ke@merck.com (X.K.); jonathan.schelfhout@merck.com (J.S.); helen.ding@merck.com (H.D.); 4 Center for Cough, Largo, FL 33778, USA; drmrsher@gmail.com; 5 Global Patient Safety, BeiGene USA, Inc., San Mateo, CA 94403, USA; lilizhou@pharmacy.arizona.edu; 6 Center for Drug Evaluation and Safety (CoDES), College of Pharmacy, University of Florida, Gainesville, FL 32610, USA

**Keywords:** cough, antitussive, opioid antitussive, benzonatate, dextromethorphan, gabapentinoid, ambulatory care visits, emergency department, NAMCS, NHAMCS

## Abstract

Using 2003–2018 National Ambulatory Medical Care Survey data for office-based visits and 2003–2018 National Hospital Ambulatory Medical Care Survey data for emergency department (ED) visits, we conducted cross-sectional analyses to examine cough medication (CM) use trends in the United States (US) ambulatory care settings. We included adult (≥18 years) patient visits with respiratory-infection-related or non-infection-related cough as reason-for-visit or diagnosis without malignant cancer or benign respiratory tumor diagnoses. Using multivariable logistic regressions, we examined opioid antitussive, benzonatate, dextromethorphan-containing antitussive, and gabapentinoid use trends. From 2003–2005 to 2015–2018, opioid antitussive use decreased in office-based visits (8.8% to 6.4%, P*_trend_* = 0.03) but remained stable in ED visits (6.3% to 5.9%, P*_trend_* = 0.99). In both settings, hydrocodone-containing antitussive use declined over 50%. Benzonatate use more than tripled (office-based:1.6% to 4.8%; ED:1.5% to 8.0%; both P*_trend_* < 0.001). Dextromethorphan-containing antitussive use increased in ED visits (1.8% to 2.6%, P*_trend_* = 0.003) but stayed unchanged in office-based visits (3.8% to 2.7%; P*_trend_* = 0.60). Gabapentinoid use doubled in office-based visits (1.1% in 2006–2008 to 2.4% in 2015–2018, P*_trend_* < 0.001) but was negligible in ED visits. In US office-based and ED ambulatory care settings, hydrocodone-containing antitussive use substantially declined from 2003 to 2018, while benzonatate use more than tripled, and dextromethorphan-containing antitussive and gabapentinoid use remained low (<3%).

## 1. Introduction

Cough is one of the most frequently reported reasons for seeking medical attention in the United States (US) [1,2]. Cough can be caused or triggered by a variety of respiratory or non-respiratory disorders and/or environmental irritants (e.g., air pollution, cigarette smoke) [3]. The 2006 American College of Chest Physicians (ACCP) clinical practice guidelines classify cough based on its duration into acute (<3 weeks), subacute (3–8 weeks), or chronic (>8 weeks) cough [4]. An empiric integrative approach is recommended to the diagnosis and management of cough based on the duration of the cough, primarily focusing on the exploration of potential causes [4,5]. For chronic cough, unexplained chronic cough (UCC) refers to when chronic cough remains unexplained without a clear etiology despite extensive clinical evaluation for common and uncommon causes, and refractory chronic cough (RCC) refers to when chronic cough remains persistent after receiving appropriate diagnostic work-up and guideline-based cough management for underlying conditions (e.g., asthma, upper airway cough syndrome, gastroesophageal reflux disease, or nonasthmatic eosinophilic bronchitis) [6,7,8].

Medications commonly used as cough suppressants include opioid antitussives, with or without antihistamines, nasal decongestants, or expectorants [9,10]. The evidence supporting the widespread use of opioid antitussives for all types of coughs is limited and has inconsistent findings [11]. The 2006 ACCP clinical practice guidelines for cough management recommends codeine-containing antitussives for chronic bronchitis based on fair-quality evidence [4]. However, the quality of evidence is low for the use of codeine-containing antitussives for patients with subacute postinfectious cough unresponsive to other treatments, and for the use of pholcodine-, hydrocodone-, dihydrocodeine-, or morphine-containing antitussives for lung cancer-related cough [4,12]. Furthermore, opioid antitussives have been shown to be ineffective in suppressing acute cough associated with upper respiratory tract infections (URTIs) [13,14] and currently there is no guideline recommendation for using opioid antitussives for RCC/UCC [6]. This inconsistent evidence on treatment effects of opioid antitussives for different types of cough could be attributed to different pathophysiologic mechanisms of cough initiation and suppression between acute and chronic cough [15]. More recently, emerging evidence indicates that cough hypersensitivity syndrome may be involved in various cough-related conditions, including RCC/UCC, although cough hypersensitivity syndrome might not be directly measurable in claims or electronic health records (EHR) structured data [16]. Gabapentinoids may suppress cough hypersensitivity syndrome [17,18], and thus gabapentin is suggested by the 2016 CHEST guidelines as a potential treatment for UCC when risks and benefits of gabapentin therapy were carefully evaluated at initiation and reassessed at 6 months before continuing gabapentin [6]. However, gabapentinoid use for RCC/UCC is off-label and not approved by US Food and Drug Administration. Potential abuse and overdose risk of gabapentinoids have also raised safety concerns for its wide off-label use [19,20,21,22,23].

In the midst of the US opioid crisis, numerous policies have been implemented to reduce potentially unsafe opioid prescribing in the past decade [24]. However, codeine- or hydrocodone-containing antitussives are typically excluded from studies in the published literature under the assumptions of their low morphine milligram equivalence doses, short-term use, and low risk of addiction and overdose. Meanwhile, widespread off-label use of gabapentinoids has raised safety concerns regarding their potential abuse and overdose risks. Little is known about the patterns of cough medication (CM) use over time in light of these factors.

In this study, we aimed to examine the trends in CM use, as well as patient, visit, and practice characteristics in the use of opioid antitussives, benzonatate, dextromethorphan-containing antitussives, and gabapentinoids among cough-related visits in US office-based ambulatory care settings and emergency departments (EDs) using nationally representative data. We hypothesized that the use of opioid antitussives has decreased due to multiple opioid prescribing restrictions, while non-opioid CM use has increased.

## 2. Materials and Methods

### 2.1. Study Design and Data Sources

We conducted repeated annual cross-sectional analyses to (1) quantify the annual proportion of cough-related visits among all eligible adult visits, (2) estimate the annual proportion of visits reporting CM use among cough-related visits, and (3) examine the trends in those annual proportions over time. For these analyses, we used the publicly available National Ambulatory Medical Care Survey (NAMCS) data from 2003 to 2018 (excluding 2017 due to data unavailability) and the ED component of the National Hospital Ambulatory Medical Care Survey (NHAMCS) data from 2003 to 2018. The NAMCS and NHAMCS data are nationally representative samples of US office-based and hospital-based ambulatory care settings, respectively [25]. NAMCS applies a three-stage probability sampling design consisting of primary sampling units, physician practices, and patient visits. A sample of physicians from the American Medical Association and American Osteopathic Association master files are asked to report patient visits during a randomly selected one-week period within a year. The NAMCS sample selection criteria exclude physicians in anesthesiology, pathology, and radiology specialties. NAMCS collects information including patients’ sociodemographics, diagnoses, medication use, prescriber specialties, and type of insurance coverage. The medication data include both prescription and over-the-counter drugs administered, ordered, continued, or supplied during each visit. Similar to NAMCS, NHAMCS uses a three-stage probability sampling design to randomly select a representative sample of ED visits over a 4-week period in non-institutional, general, and short-stay hospitals (excluding federal hospitals) in the US [26]. NHAMCS collects similar information as NAMCS. NAMCS and NHAMCS are publicly available de-identified data; thus, the University of Florida Institutional Review Board deemed its human subjects exempt from review.

### 2.2. Study Cohort

For inclusion criteria in our primary analysis, we restricted to adult (≥18 years) visits with any cough-related reason for visit or diagnosis (hereafter cough-related visits) using the first three reason-for-visit codes or diagnosis codes (identified by the International Classification of Diseases, Ninth Revision, Clinical Modification [ICD-9-CM]/ICD-10-CM codes) (Table S1). The ICD-9/10-CM code for cough is found in the signs and symptoms section of the ICD-9/10-CM official guidelines for coding and reporting. The guidance for coding this section is to not code signs and symptoms of a disease process as additional diagnoses [27,28]. Thus, we included visits with respiratory conditions commonly accompanied by cough (i.e., URTIs, chronic upper respiratory tract diseases, influenza, bronchitis, pneumonia) and did not restrict to visits with cough-specific codes. NAMCS and NHAMCS collected up to three reason-for-visit codes and diagnosis codes prior to 2014 and up to five reason-for-visit codes and diagnosis codes after 2014 for each visit. To ensure findings are comparable across years, our primary analysis was restricted to the first three reason-for-visit codes and diagnosis codes, which align with previously published NAMCS/NHAMCS studies (See relevant sensitivity analyses in Section 2.6) [29]. For our exclusion criteria, we excluded visits with any malignant cancer diagnoses or any benign respiratory tumors because cough caused by these conditions and their related treatments (e.g., chest radiation) may be different from general cough conditions. In addition, cancer patients are more likely to be prescribed opioids for pain than non-cancer patients. Opioid analgesic medications also have cough suppression effects, which in turn might make cancer patients less likely to be prescribed additional opioid antitussives. We did not exclude any approved indications of gabapentinoids, since most of the gabapentinoid use (>96%) was off-label in ambulatory care settings based on a prior NAMCS study [29]. Eligible cough-related visits served as the final analytical sample and denominator for estimating the annual proportion of visits reporting CM use among cough-related visits for each year.

The overall visit characteristics of NAMCS and NHAMCS data applying the above inclusion and exclusion criteria in 2003–2018 were as follow: age younger than 65 years (NAMCS: 67.2% vs. NHAMCS: 80.1%), female (61.2% vs. 57.1%), White (84.6% vs. 73.5%), having <2 chronic conditions (37.3%% vs. 26.6%), and residing in Northeastern region (19.7% vs. 18.3%), Midwest region (20.4% vs. 23.1%), Southern region (37.9% vs. 38.7%), and West region (22.1% vs. 19.9%).

### 2.3. Medications

Among cough-related visits, we identified the visits with the following CMs prescribed, supplied, administered, or continued: (1) opioid-containing combinations subclassified as codeine, dihydrocodeine, and hydrocodone or codeine monotherapy (hereafter opioid antitussives); (2) benzonatate; (3) dextromethorphan-containing antitussives; and (4) gabapentinoids (i.e., gabapentin and pregabalin). Opioid-containing combinations and dextromethorphan-containing antitussives may include an expectorant (e.g., guaifenesin), an H1 antihistamine (e.g., brompheniramine, chlorpheniramine), or a decongestant (e.g., phenylephrine, pseudoephedrine). We ascertained medications of interest using the Multum Lexicon Plus^®^ System (Table S2). As a reference to national trends in overall opioid use, we included a comparison group of visits that reported the use of opioid analgesics (i.e., codeine, hydrocodone, dihydrocodeine, morphine, oxycodone, hydromorphone, and oxymorphone classified as ‘060: narcotic analgesics’ or ‘191: narcotic analgesic combination’) (Table S2). Data collection for the number of medications prescribed, supplied, administered, or continued varied across years (i.e., up to 8 medications prior to 2011, 10 medications in NAMCS/12 medications in NHAMCS in 2012–2013, and 30 medications after 2014). Consistent with prior published literature, our primary analysis was restricted to the first eight medication codes listed to ensure the findings were comparable across years [29].

### 2.4. Patient, Visit, and Practice Characteristics

For both NAMCS and NHAMCS analyses, we extracted patient characteristics including age at ambulatory care visit (18–64 years or ≥65 years), sex (female or male), and race (White or non-White). Visit characteristics included: payment source (governmental insurance including Medicare and Medicaid, commercial insurance, or others) and number of chronic conditions (<2 or ≥2). The value for the number of chronic conditions variable was calculated based on the presence of a particular set of chronic conditions included in the NAMCS/NHAMCS survey questionnaires. The practice location characteristics included geographic region (Northeast, Midwest, South, or West) and metropolitan status (metropolitan or non-metropolitan). To characterize the visits included in this study, we included the most common three reasons-for-visit and three diagnoses for both NAMCS and NHAMCS. NAMCS data provided additional patient, visit, and practice characteristics that were included in this analysis: smoking status (current or non-concurrent smokers), the chronicity of the principal reason for visit (chronic conditions [routine or flare-up] or others), and prescriber specialty. NAMCS stratifies prescriber specialties into 15 categories: general and family practice, internal medicine, pediatrics, general surgery, obstetrics and gynecology, orthopedic surgery, cardiovascular diseases, dermatology, urology, psychiatry, neurology, ophthalmology, otolaryngology, oncology, and others. We categorized specialties into primary care providers (including general/family practice and internal medicine) and others based on the distribution of the data in our analysis.

### 2.5. Statistical Analysis

We first quantified the proportion of cough-related visits among all eligible adult visits. Next, we estimated the annual proportion of visits reporting the use of CMs among cough-related visits using complex survey design procedures (i.e., SURVEYFREQ and SURVEYLOGISTIC) in SAS version 9.4 (SAS Institute Inc, Cary, NC, USA). Sampling weights from NAMCS/NHAMCS were applied to obtain national weighted-estimates. Two-sided *p*-values less than 0.05 were considered statistically significant. The National Center for Health Statistics (NCHS) recommends excluding the results from analyses for unweighted count values less than 30 or when the relative standard error is greater than 30% due to the unreliability of these estimates [30]. The results from the trend analyses of gabapentinoid use in NHAMCS (except in all eligible adult visits) and the associated patient, visit, and practice characteristics were, thus, not presented in this study. To obtain reliable estimates, we aggregated annual data into five consecutive time periods. Notably, the last grouping in NAMCS for 2015–2018 did not include information for 2017 as data are not available. We compared the characteristic differences between five medication groups (i.e., opioid antitussives, benzonatate, dextromethorphan-containing antitussives, gabapentinoids, and opioid analgesics) using absolute standardized differences (ASD), where an absolute ASD > 0.1 indicates a significant difference [31]. We used multivariable logistic regression to test the significance of trends in (1) the number of cough-related visits among all eligible adult visits, and (2) the number of opioid antitussives, benzonatate, dextromethorphan-containing antitussives, gabapentinoids, and opioid analgesics used among cough-related visits over time. We adjusted for age, sex, race, and payment source in both NAMCS and NHAMCS analyses.

### 2.6. Secondary and Sensitivity Analyses

We conducted two secondary analyses using different definitions of denominators for CM trend analyses to provide a context for our primary analysis since there is no definitive standard for identifying cough-related visits: (1) cough-specific visits identified using a reason-for-visit code of 1440.0 or diagnosis of cough (ICD-9-CM code: 786.2; ICD-10-CM code: R05), and (2) all eligible adult visits. Our primary trend analysis only used the first three reason-for-visit codes and diagnosis codes, and the first eight medication codes listed. This approach may underestimate the number of cough-related visits and medication use in the later years of our study period. Thus, to ensure the robustness of our findings, we also conducted sensitivity analyses including: (1) all available medication codes listed, (2) all available reason-for-visit codes and diagnosis codes listed, and (3) all available medication codes, reason-for-visit codes, and diagnosis codes listed for each patient visit during the study period.

## 3. Results

### 3.1. NAMCS Analysis: 2003–2018

#### 3.1.1. Patient, Visit, and Practice Characteristics

Table 1 shows the patient, visit, and practice characteristics for different medication groups of interest among cough-related office-based visits from 2003 to 2018. Among a weighted-estimate of 11.1 billion adult office-based ambulatory visits from 2003 to 2018, we identified 819.9 million (7.4%) cough-related visits. Among cough-related office-based visits from 2003 to 2018, the overall prevalence of medication use was 7.7% for opioid antitussives, 2.5% for benzonatate, 3.6% for dextromethorphan-containing antitussives, 1.6% for gabapentinoids, and 5.0% for opioid analgesics. The majority of the cough-related office-based visits with opioid antitussives were from patients who were younger than 65 years (76.5%), female (64.8%), White (81.0%), non-current smokers (81.5%), and having <2 chronic conditions (60.3%). Overall, the characteristics of cough-related office-based visits with benzonatate or dextromethorphan-containing antitussives were similar to visits involving opioid antitussives. Contrastingly, cough-related office-based visits with gabapentinoids were more likely from patients aged ≥65 years (41.2%), having a chronic condition as a principal reason-for-visit (37.8%), and having ≥2 chronic conditions (64.8%). The most common payment source for cough-related office-based visits with opioid antitussives was commercial insurance (62.7%), followed by governmental insurance (27.7%), whereas 53.2% of cough-related office-based visits with gabapentinoids were paid by governmental insurance. The most common two diagnoses (other than a cough-specific diagnosis) associated with antitussive use were bronchitis and acute URTI. Over 75% of cough-related office-based visits with antitussives were seen by primary care physicians. In general, over 40% of these medication uses were observed in the Southern region of the US (opioid antitussives: 44.6%; benzonatate: 53.8%; dextromethorphan-containing antitussives: 42.0%; gabapentinoids: 43.1%). Specifically, 62.6% of cough-related office-based visits with hydrocodone-containing antitussive use and 27.1% of cough-related office-based visits with codeine-containing antitussive use were observed in the US Southern region (Table S3).

#### 3.1.2. Trends in Cough-Related Office-Based Visits and Medication Use

**Figure 1:**
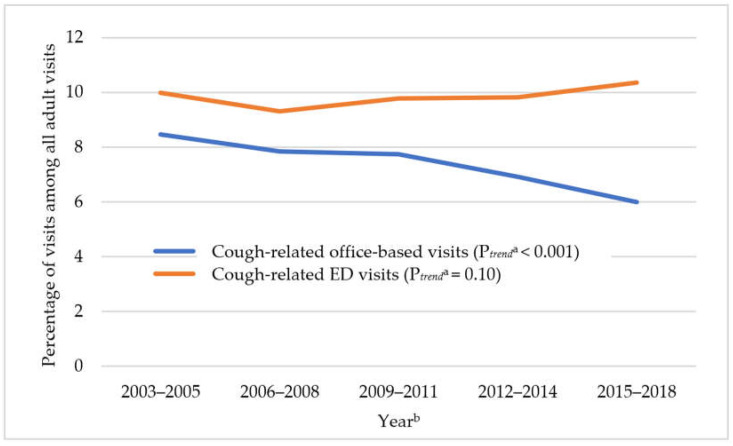
Trends in prevalence of adult cough-related visits among all adult visits in US ambulatory care settings: 2003–2018 NAMCS and 2003–2018 NHAMCS data. Abbreviations: NAMCS: National Ambulatory Medical Care Survey; NHAMCS: National Hospital Ambulatory Medical Care Survey; ED: Emergency Department. ^a^ P*_trend_* were adjusted for age, sex, race, and payment source. ^b^ The weighted-estimate of the denominator (i.e., all adult visits) for each time period in NAMCS is 2.2 billion, 2.2 billion, 2.3 billion, 2.2 billion, 2.2 billion for 2003–2005, 2006–2008, 2009–2011, 2012–2014, and 2015–2018, respectively. The weighted-estimate of the denominator (i.e., all adult visits) for each time period in NHAMCS is 252.9 million, 277.0 million, 308.1 million, 312.7 million, and 421.5 million for 2003–2005, 2006–2008, 2009–2011, 2012–2014, and 2015–2018, respectively.

**Figure 2:**
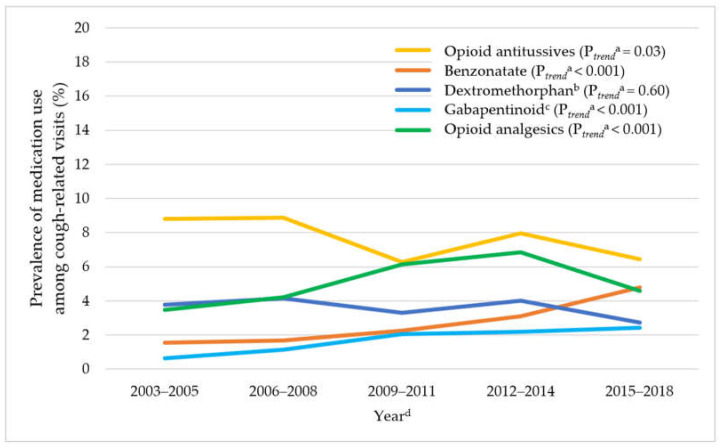
Trends in medication use among adult cough-related visits in office-based ambulatory care setting: 2003–2018 NAMCS. Abbreviations: NAMCS: National Ambulatory Medical Care Survey; NCHS: National Center for Health Statistics. ^a^ P_trend_ were adjusted for age, sex, race, and payment source. ^b^ Dextromethorphan indicates dextromethorphan-containing antitussives. ^c^ The weighted-estimate for gabapentinoid in 2003–2005 is unreliable based on NCHS’s recommendation. ^d^ The weighted-estimate of the denominator (i.e., adult cough-related office-based visits) for each time period in NAMCS is 183.7 million, 171.9 million, 179.7 million, 152.8 million, and 131.8 million for 2003–2005, 2006–2008, 2009–2011, 2012–2014, and 2015–2018, respectively.

In Figure 1, cough-related office-based visits declined by 29.3% over time from 8.5% in 2003–2005 to 6.0% in 2015–2018 (P*_trend_* < 0.001). As shown in Figure 2, among cough-related office-based visits, there were significant increasing trends in benzonatate use (from 1.6% in 2003–2005 to 4.8% in 2015–2018, P*_trend_* < 0.001), and gabapentinoid use (from 1.1% in 2006–2008 to 2.4% in 2015–2018, P*_trend_* < 0.001), while opioid antitussive use decreased (from 8.8% in 2003–2005 to 6.4% in 2015–2018, P*_trend_* = 0.03) and dextromethorphan-containing antitussive use remained stable (from 3.8% in 2003–2005 to 2.7% in 2015–2018, P*_trend_* = 0.60). Opioid analgesic use increased from 2003–2005 to 2012–2014 and then decreased (3.5% in 2003–2005, 6.9% in 2012–2014, and 4.6% in 2015–2018, P*_trend_* < 0.001). The decreasing trend in opioid antitussive use was largely driven by the reduced prevalence of hydrocodone-containing antitussive use from 5.2% in 2003–2005 to 3.3% in 2012–2014, P*_trend_* < 0.001) (Figure S1).

### 3.2. NHAMCS Analysis: 2003–2018

#### 3.2.1. Patient, Visit, and Practice Characteristics

Table 2 shows the patient, visit, and practice characteristics for different medication groups of interest among cough-related ED visits from 2003 to 2018. Among a weighted-estimate of 1.6 billion adult ED visits from 2003 to 2018, we identified 155.5 million (9.9%) cough-related ED visits. Among cough-related ED visits from 2003 to 2018, the overall prevalence of medication use was 6.2% for opioid antitussives, 4.8% for benzonatate, 1.9% for dextromethorphan-containing antitussives, and 14.6% for opioid analgesics. The majority of the cough-related ED visits with antitussives (i.e., opioid antitussives, benzonatate, dextromethorphan-containing antitussives) were from patients who were younger than 65 years (range: 89.2–89.5%), female (range: 62.1–65.8%), and having <2 chronic conditions (72.2–75.1%). The most common payment source for cough-related ED visits with antitussives was governmental insurance (range: 37.8–40.7%), followed by commercial insurance (26.6–31.9%). The most common two diagnoses (other than a cough-specific diagnosis) associated with antitussive use were bronchitis and acute URTI. Over 75% of cough-related ED visits with antitussives were from metropolitan areas. Specifically, 66.1% of cough-related ED visits with hydrocodone-containing antitussive use and 41.5% of cough-related ED visits with codeine-containing antitussive use were observed in the US Southern region (Table S4). The results from the stratification analysis of gabapentinoids in NHAMCS were not presented in this study due to the small number of unweighted visits.

#### 3.2.2. Trends in Cough-Related ED Visits and Medication Use

**Figure 3:**
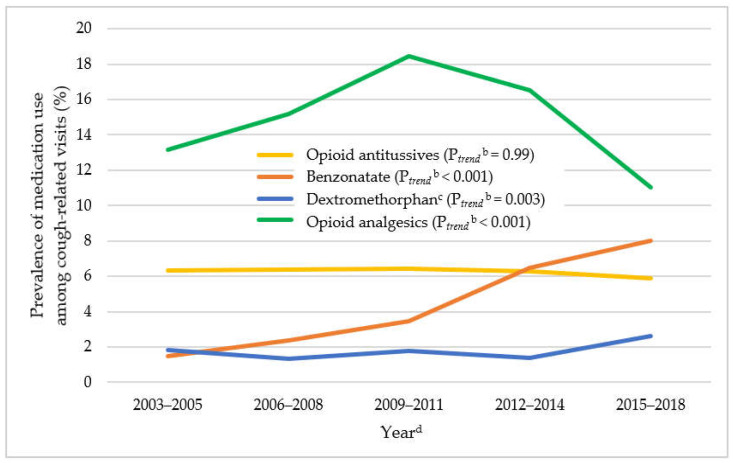
Trends in medication use among adult cough-related visits in ED setting: 2003–2018 NHAMCS^a^. Abbreviations: NHAMCS: National Hospital Ambulatory Medical Care Survey; ED: Emergency Department; NCHS: National Center for Health Statistics. ^a^ The trend for gabapentinoids is not included because it had an unweighted count value of less than 30 in each time period, which is unreliable based on NCHS’s recommendation. ^b^ P*_trend_* were adjusted for age, sex, race, and payment source. ^c^ Dextromethorphan indicates dextromethorphan-containing antitussives. ^d^ The weighted-estimate of the denominator (i.e., adult cough-related ED visits) for each time period in NHAMCS is 25.2 million, 25.8 million, 30.1 million, 30.7 million, and 43.7 million for 2003–2005, 2006–2008, 2009–2011, 2012–2014, and 2015–2018, respectively.

Cough-related ED visits remained unchanged (from 10.0% in 2003–2005 to 10.4% in 2015–2018, P*_trend_* = 0.10) (Figure 1). Among cough-related ED visits from 2003 to 2018, the overall prevalence of medication use was 6.2% for opioid antitussives, 4.8% for benzonatate, 1.9% for dextromethorphan-containing antitussives, 0.3% for gabapentinoids, and 14.6% for opioid analgesics. As shown in Figure 3, among cough-related ED visits, there were significant increasing trends in benzonatate use (from 1.5% in 2003–2005 to 8.0% in 2015–2018, P*_trend_* < 0.001), while opioid antitussive use stayed stable (from 6.3% in 2003–2005 to 5.9% in 2015–2018, P*_trend_* = 0.99). A decreasing trend in hydrocodone-containing antitussive use (from 3.8% in 2003–2005 to 1.8% in 2015–2018, P*_trend_* = 0.004) was offset by an increasing trend in codeine-containing antitussive use (2.6% in 2003–2005 to 4.1% in 2015–2018, P*_trend_* = 0.008, Figure S2). Opioid analgesic use reached a peak in 2009 through 2011 and then decreased over time (13.1% in 2003–2005, 18.4% in 2009–2011, and 11.0% in 2015–2018, P*_trend_* < 0.001). The results from the trend analysis of gabapentinoids in NHAMCS were not presented in this study due to the small number of unweighted visits across the years.

### 3.3. Secondary and Sensitivity Analyses

Cough-specific office-based visits decreased from 3.6% in 2003–2005 to 2.7% in 2015–2018 (P*_trend_* = 0.01), while cough-specific ED visits increased from 3.7% in 2003–2005 to 5.5% in 2015–2018 (P*_trend_* < 0.001) (Figure S3). The trends in the prevalence of medication use among cough-specific visits are consistent with those among cough-related visits in the primary analysis, with an overall high prevalence due to the deflation in the denominators (Figures S4 and S5). The prevalence of antitussive use (opioid antitussives, benzonatate, dextromethorphan-containing antitussives) among all adult visits was <1% in both settings due to the inflation in the denominators (Figures S6 and S7). Sensitivity analyses using different numbers of medication and/or reason-for-visit and diagnosis variables yielded similar findings to those reported in the primary NAMCS and NHAMCS analyses (data not shown).

## 4. Discussion

Using nationally representative office-based ambulatory care (NAMCS) and ED (NHAMCS) visit data, our study yielded three important findings. First, there was a decreasing trend in cough-related visits in office-based ambulatory care settings, while cough-related visits in ED settings stayed stable. Second, the prevalence of antitussive use among cough-related visits over time was generally low (<9%), regardless of being either an office-based or ED setting. Notably, cough-related visits with hydrocodone-containing antitussives (with a higher risk of overdose and addiction compared to codeine) declined whereas cough-related visits with benzonatate increased substantially from 2003–2005 to 2015–2018 in both settings. Furthermore, we found similarities in patient, visit, and practice characteristics between the cough-related visits with reported use of opioid antitussives, benzonatate, and dextromethorphan-containing antitussives. Finally, our findings indicated room for improvement in opioid antitussive use based on the gap between the available evidence and clinical practice, such as opioid antitussive use for URTI-related visits that are not supported by evidence. We also identified regional variations in opioid antitussive use among cough-related visits that require further investigation to understand the reasons.

The decreasing trend in cough-related office-based visits in our study may be partially explained by improvement in patient awareness and perceptions about appropriate antibiotic use for URTIs and their self-management of URTIs, most of which do not require office visits nor the use of antibiotics or other prescriptions [32]. However, the decrease in cough-related office-based visits did not affect the cough-related ED visits, which accounted for approximately 10% of overall adult ED visits during the study period. A study of 2016 data using Healthcare Cost and Utilization Project’s (HCUP) National Emergency Department Sample (NEDS) data, found that the second most common reason for ED visits was URTIs [33]. Although the NHAMCS data do not include urgent care centers, the decrease in cough-related office-based visits may be attributable to an increasing trend in urgent care use. A recent study reported a 32.5% increase in urgent care visits with respiratory conditions from 2008 to 2015 [34].

Although the prevalence of antitussive use among cough-related visits over time was generally low (<9%) in ambulatory care settings, opioid antitussives were the most commonly prescribed type of cough-suppressing medication in office-based ambulatory care settings across our study period. It is likely that the decreasing trend in cough-related visits with hydrocodone-containing antitussives was influenced by numerous opioid-related policies implemented in recent years to prevent unsafe opioid prescription use and an increase in clinicians’ awareness of opioid-related adverse outcomes. It appears that the decreasing trend in hydrocodone-containing antitussive use was offset by an increase in codeine-containing antitussive use in ED settings, while a decrease in hydrocodone-containing antitussive use did not impact codeine-containing antitussive use in office-based ambulatory care settings. Rather, hydrocodone-containing antitussives appear to have been largely substituted with benzonatate in office-based ambulatory care settings.

The changes in antitussive prescribing patterns that we found reflect the characteristics of the patients visiting in each setting. The severity and urgency of cases seen in ED settings may still require the substitution of opioid antitussives (i.e., codeine-containing antitussives) for hydrocodone-containing antitussives. Furthermore, these changes were consistent with the observed trends in general opioids prescribed at ED discharge in the US, which indicated a decrease in the use of opioids with stronger potency (e.g., acetaminophen-hydrocodone) and an increase in the use of opioids with weaker potency (e.g., acetaminophen-codeine) from 2010 to 2017 [35]. Another potential explanation could be that the 2006 ACCP guidelines explicitly recommend codeine-containing antitussives for short-term symptomatic relief in postinfectious and chronic bronchitis-related cough [4]. Given that the Centers of Disease Control and Prevention (CDC) guidelines for opioid prescribing were released towards the end of our study period, and there is extensive evidence showing overall national decreases in opioid prescribing in the US, the decreasing trend observed in this study is not likely to be reversed in data from more recent years.

Indeed, benzonatate and gabapentinoid use steeply increased in our samples. Benzonatate use nearly tripled among cough-related visits in office-based ambulatory care settings and more than quintupled among cough-related visits in ED settings from 2003 to 2018. In addition, gabapentinoid use more than doubled among cough-related visits in office-based ambulatory care settings from 2006 to 2018. The magnitude of the increase (i.e., doubled) in gabapentinoid use observed in our study was smaller than the four-fold increase reported in a prior study focusing on general adult ambulatory visits from 2003 to 2016 using NAMCS data [29]. Recent case reports [36,37], case series [38,39,40], chart reviews [41,42], and randomized controlled trials (RCTs) [17,18] reported gabapentinoids as a potential pharmacological option for UCC [43]. In 2016, the CHEST guidelines for UCC management suggested gabapentin could be considered for UCC with careful benefit-risk assessment by a cough specialist at initiation and its continuation when the positive benefit-risk balance remains at 6 months [6]. Given a low prevalence of UCC among populations with cough [44], and a high prevalence of gabapentinoid use (3.9% in 2015) in the general population [29,45], it is hard to conclude that the observed increasing trend in gabapentinoid use among cough-related visits in our data is ascribed to their use for treating UCC. Further studies are warranted to estimate prescribing prevalence of gabapentinoids and to evaluate the risk-benefit assessment of gabapentinoid use among patients with UCC, especially its use after the publication of the CHEST guidelines in 2016 [6]. 

We identified several substantial gaps or discrepancies in opioid antitussive use for cough between the available evidence and clinical practice. Nearly one-third of cough-related visits with opioid antitussive prescriptions had a diagnosis of acute respiratory tract infections, which do not warrant opioid antitussive use due to a lack of efficacy reported in prior RCTs [13,14]. In a double-blind RCT conducted by Eccles et al., there was no difference in the reduction of cough frequency and severity between codeine and placebo groups [13]. Similar results were reported in Freestone et al.’s study using three different measures of cough [14]. Given a lack of clinical evidence and potential adverse outcomes, the risks may outweigh the benefits of opioid antitussive use for suppressing URTI-associated cough. Furthermore, more than 60% of cough-related visits with hydrocodone-containing antitussive use were concentrated in the US Southern region. Hydrocodone-containing antitussives were more commonly used opioid antitussives than codeine-containing antitussives in the South as opposed to other US regions. This indicates that regional variations in opioid antitussive prescribing may exist. Additional research is needed to examine the reasons (e.g., patient or physician prescribing preferences) for these regional variations in opioid antitussive use.

Even though this is the first study to our knowledge examining trends in CM use in US ambulatory care settings using nationally representative data over several years, there are several limitations to acknowledge when interpreting the findings. First, NAMCS/NHAMCS are visit-level data that do not allow longitudinal patient follow-up to identify the type of cough (i.e., acute, subacute, and chronic), and they do not contain information on cough severity or medication dose and duration of use. Second, we used the limited number of ICD-9/10-CM diagnosis codes and reason-for-visit codes available in NAMCS/NHAMCS to identify cough-related visits. Given that cough can be considered a mild ailment when multiple chronic comorbidities are present, this may underestimate the number of cough-related visits. Finally, although NAMCS/NHAMCS capture over-the-counter dextromethorphan-containing antitussive use as well as prescription dextromethorphan-containing antitussives, there is a potential underestimation of over-the-counter drug use, since it merely relies on patients’ reports to a physician at the visits. Given that over 97% of gabapentinoid use was off-label [29], the upward trend in gabapentinoid use among cough-related visits in office-based ambulatory care settings may not necessarily reflect its use for cough-related conditions.

## 5. Conclusions

Among cough-related visits in the US ambulatory care settings from 2003 to 2018, cough-related visits with antitussive (i.e., opioid antitussives, benzonatate, dextromethorphan) use were generally low (<9%), regardless of being in office-based or ED settings. Hydrocodone-containing antitussive use decreased by over 50%, while benzonatate use increased in conjunction with the initiation of stricter policies and regulations regarding opioid prescribing. Moreover, the decreasing trend in hydrocodone-containing antitussive use was offset by an increase in codeine-containing antitussive use among cough-related ED visits. There were significant regional variations in CM use, particularly over 60% of cough-related visits with hydrocodone-containing antitussive use were concentrated in the US Southern region. This study suggests that further examination of the use of these drugs in each subtype of cough or cough-related condition is still warranted to assess the effects of these changing trends.

## Figures and Tables

**Table 1:** Patient, visit, and practice characteristics of adult cough-related office-based visits by medication type: 2003–2018 NAMCS (weighted-estimate of 819.9 million).

Characteristics	Opioid Antitussives	Benzonatate	Dextromethorphan ^a^	Gabapentinoids	Opioid Analgesics	Mean ASD ^b^
	63.3 Million (7.7%)	20.9 Million (2.5%)	29.7 Million (3.6%)	13.3 Million (1.6%)	41.1 Million (5.0%)	
	Weighted %	Weighted %	Weighted %	Weighted %	Weighted %	
Patient Characteristics						
Age ≥ 65 years	23.5	25.7	21.5	41.2	24.7	0.21
Female	64.8	71.3	62.6	62.9	61.6	0.09
Race						0.10
White	81.0	84.1	78.9	87.5	83.1	
Non-White	19.0	15.9	21.1	12.5	16.9	
Smoking status ^c^						0.16
Current	15.3	12.9	14.8	19.7	24.6	
Non-current	81.5	83.0	81.3	78.0	72.0	
Visit Characteristics						
Payment source ^d^						0.33
Governmental	27.7	30.4	31.7	53.2	44.6	
Commercial	62.7	60.1	54.9	37.3	45.5	
Others	7.5	6.3	11.4	5.0	7.7	
Chronicity of principal reason-for-visit ^e^	12.9	15.5	12.0	37.8	34.1	0.37
≥2 Chronic conditions ^f^	39.7	41.1	30.7	64.8	45.5	0.29
Top 3 major reasons-for-visit						
	Cough 65.1	Cough 64.0	Cough 55.3	Cough 38.3	Cough 39.8	
	Nasal congestion 21.4	Nasal congestion 18.3	Throat symptom 26.0	Nasal congestion 12.5	Throat symptom 14.5	
	Throat symptom 19.0	Throat symptom 16.7	Nasal congestion 22.9	Throat symptom 11.1	Nasal congestion 10.7	
Top 3 major diagnoses						
	Bronchitis 39.1	Acute URTI 39.8	Acute URTI 53.4	Bronchitis 22.6	Acute URTI 30.8	
	Acute URTI 36.8	Cough 30.0	Bronchitis 27.0	Acute URTI 21.9	Bronchitis 25.8	
	Cough 15.8	Bronchitis 26.1	Cough 14.9	Chronic URTD 17.7	Chronic URTD 16.4	
Practice characteristics						
Prescriber specialty						0.37
Primary care	88.7	89.0	88.6	76.7	80.1	
Others	11.3	11.0	11.4	23.3	19.9	
Geographic region ^g^						0.22
Northeast	12.4	11.1	21.7	12.4	10.9	
Midwest	19.5	16.2	14.4	22.4	20.9	
South	44.6	53.8	42.0	43.1	41.4	
West	23.5	19.0	21.8	22.1	26.9	
Metropolitan area	86.7	90.9	88.5	76.4	83.6	0.14

Abbreviations: NAMCS: National Ambulatory Medical Care Survey; ASD: absolute standardized difference; URTI: upper respiratory tract infections; URTD: upper respiratory tract diseases. ^a^ Dextromethorphan indicates dextromethorphan-containing antitussives. ^b^ ASD > 0.1 was considered as having non-negligible differences. Represented the mean ASD across the 10 ASDs from all possible two-pairs group comparisons (e.g., opioid antitussives vs. benzonatate). ^c^ The percentage of missingness for smoking status among cough-related office-based visits involving five medication groups were ≤5.4% from 2003 to 2018. Non-concurrent smoker includes never smoker, former smoker, and unknown. ^d^ The percentage of missingness for payment source among cough-related office-based visits involving five medication groups were ≤2.5% from 2003 to 2018. Others include all other types of insurance, uninsured, and unknown. ^e^ The percentage of missingness for chronicity of major reason-for-visit among cough-related office-based visits involving five medication groups were ≤1.3% from 2003 to 2018. ^f^ The number of chronic conditions was available in NAMCS since 2005. The percentage of missingness for the variable of ≥2 chronic conditions among cough-related office-based visits involving five medication groups were ≤1.7% from 2005 to 2018. ^g^ The region was unavailable in NAMCS in 2018.

**Table 2:** Patient, visit, and practice characteristics of adult cough-related ED visits by medication type: 2003–2018 NHAMCS (weighted-estimate of 155.5 million).

Characteristics	Opioid Antitussives	Benzonatate	Dextromethorphan ^a^	Opioid Analgesics	Mean ASD ^b^
	9.7 Million (6.2%)	7.5 Million (4.8%)	2.9 Million (1.9%)	22.7 Million (14.6%)	
	Weighted%	Weighted%	Weighted%	Weighted%	
Patient Characteristics					
Age ≥ 65 years	10.6	10.8	10.5	15.4	0.07
Female	63.2	65.8	62.1	63.0	0.03
Race					0.13
White	71.3	68.0	59.1	72.7	
Non-White	28.7	32.0	40.9	27.3	
Visit Characteristics					
Payment source ^c^					0.10
Governmental	37.8	40.2	40.7	46.1	
Commercial	31.9	31.5	26.6	27.8	
Others	28.6	27.7	31.5	24.5	
≥2 Chronic Conditions ^d^	24.9	26.5	27.8	31.9	0.08
Top 3 major reasons-for-visit					
	Cough 66.5	Cough 68.3	Cough 61.0	Cough 35.3	
	Throat symptoms 17.8	Throat symptoms 18.1	Throat symptoms 21.3	Throat symptoms 16.8	
	Fever 15.9	Nasal congestion 17.7	Nasal congestion 20.7	Shortness of breath 15.0	
Top 3 major diagnoses					
	Bronchitis 44.6	Bronchitis 40.9	Acute URTI 37.0	Acute URTI 28.4	
	Acute URTI 28.8	Acute URTI 33.3	Bronchitis 34.5	Bronchitis 24.7	
	Cough 14.4	Cough 23.1	Cough 16.3	Pneumonia 18.6	
Practice Characteristics					
Geographic region					0.21
Northeast	12.8	16.0	9.5	10.0	
Midwest	20.0	23.0	19.8	22.6	
South	51.3	47.8	54.3	42.2	
West	15.9	13.2	16.4	25.2	
Metropolitan area ^e^	77.8	83.2	78.2	86.2	0.13

Abbreviations: NHAMCS: National Hospital Ambulatory Medical Care Survey; ASD: absolute standardized difference; URTI: upper respiratory tract infections. ^a^ Dextromethorphan indicates dextromethorphan-containing antitussives. ^b^ ASD > 0.1 was considered as having non-negligible differences. Represented the mean ASD across the six ASDs from all possible two-pairs group comparisons (e.g., opioid antitussives vs. benzonatate). ^c^ The percentage of missingness for payment source among cough-related ED visits involving four medication groups were ≤1.7% from 2003 to 2018. Others include all other types of insurance, uninsured, and unknown. ^d^ The number of chronic conditions was available since 2012. The percentage of missingness for the variable of ≥2 chronic conditions among cough-related ED visits involving four medication groups were ≤1.1% from 2012 to 2018. ^e^ The metropolitan area was unavailable in NHAMCS in 2012.

## Data Availability

The data presented in this study are openly available from the National Center for Health Statistics at https://www.cdc.gov/nchs/ahcd/datasets_documentation_related.htm (accessed on 22 June 2022).

## Supplementary Materials

The following are available online at https://www.mdpi.com/article/10.3390/jcm11133671/s1, Table S1: Reason-for-visit and ICD-9-CM/ICD-10-CM codes to identify cough-related visits, Table S2: Multum Lexicon Plus^®^ generic codes used to identify opioid antitussives, benzonatate, dextromethorphan-containing antitussives, gabapentinoids, and opioid analgesics in NAMCS/NHAMCS, Table S3: Patient, visit, and practice characteristics of visits hydrocodone- and codeine-containing antitussive use: 2003–2018 NAMCS (weighted-estimate of 819.9 million), Table S4: Patient, visit, and practice characteristics of visits hydrocodone- and codeine-containing antitussive use: 2003–2018 NHAMCS (weighted-estimate of 155.5 million), Figure S1: Trends in hydrocodone- and codeine-containing antitussive use among cough-related visits in office-based ambulatory care setting: 2003–2018 NAMCS, Figure S2: Trends in hydrocodone- and codeine-containing antitussive use among cough-related visits in ED setting: 2003–2018 NHAMCS, Figure S3: Trends in prevalence of adult cough-specific visits among all adult visits in US ambulatory care settings: 2003–2018 NAMCS and 2003–2018 NHAMCS data, Figure S4: Trends in medication use among cough-specific visits in office-based ambulatory care setting: 2003–2018 NAMCS, Figure S5: Trends in medication use among cough-specific visits in ED setting: 2003–2018 NHAMCS, Figure S6: Trends in medication use among all adult visits in office-based ambulatory care setting: 2003–2018 NAMCS, Figure S7: Trends in medication use among all adult visits in ED setting: 2003–2018 NHAMCS.
