# Supplementary material for: Evaluation of Cough Medication Use Patterns in Ambulatory Care Settings in the United States: 2003–2018
Source: J Clin Med. 2022 Jun 25;11(13):3671. doi: 10.3390/jcm11133671 (PMC9267927; doi:10.3390/jcm11133671)
Supplement: Supplementary file 1 [file jcm-11-03671-s001.zip › jcm-1710097-supplementary.pdf]

SUPPLEMENTARY MATERIALS

Evaluation of Cough Medication Use Patterns in Ambulatory Care Settings in the United States: 2003–2018

Seonkyeong Yang, MS; Juan M. Hincapie-Castillo, PharmD, PhD; Xuehua Ke, PhD; Jonathan Schelfhout, PhD; Helen Ding, MD, MS, MSPH; Mantel R. Sher, MD; Lili Zhou, PhD; Chang Ching-Yuan, MS; Debbie L. Wilson, PhD, RN; Wei-Hsuan Lo-Ciganic, PhD

Table S1. Reason-for-visit and ICD-9-CM/ICD-10-CM codes to identify cough-related visits

Table S2. Multum Lexicon Plus® generic codes used to identify opioid antitussives, benzonatate, dextromethorphan-containing antitussives, gabapentinoids, and opioid analgesics in NAMCS/NHAMCS

Table S3. Patient, visit, and practice characteristics of visits hydrocodone- and codeine-containing antitussive use: 2003–2018 NAMCS (weighted-estimate of 819.9 million)

Table S4. Patient, visit, and practice characteristics of visits hydrocodone- and codeine-containing antitussive use: 2003–2018 NHAMCS (weighted-estimate of 155.5 million)

Figure S1. Trends in hydrocodone- and codeine-containing antitussive use among cough-related visits in office-based ambulatory care setting: 2003–2018 NAMCS

Figure S2. Trends in hydrocodone- and codeine-containing antitussive use among cough-related visits in ED setting: 2003–2018 NHAMCS

Figure S3. Trends in prevalence of adult cough-specific visits among all adult visits in US ambulatory care settings: 2003–2018 NAMCS and 2003–2018 NHAMCS data

Figure S4. Trends in medication use among cough-specific visits in office-based ambulatory care setting: 2003–2018 NAMCS

Figure S5. Trends in medication use among cough-specific visits in ED setting: 2003–2018 NHAMCS

Figure S6. Trends in medication use among all adult visits in office-based ambulatory care setting: 2003–2018 NAMCS

Figure S7. Trends in medication use among all adult visits in ED setting: 2003–2018 NHAMCS

Table S1. Reason-for visit and ICD-9-CM/ICD-10-CM codes to identify cough-related visits

Class	Conditions	Reason-for-visit codes	ICD-9-CM codes	ICD-10-CM codes
Infection-related cough	Acute URTI	1445.0 2600.0	460 461.x 462 464.0x 464.4 465.x	J00 J01.xx J02.x J04.0 J05.0 J06.x
	Influenza	1450.0 1450.1	487.x 488.xx	J09.xx J10.xx J11.xx
	Bronchitis	2610.0	466.0 490 491.xx	J20.x J40 J41.x J42
	Pneumonia	2630.0	480.x 481 482.xx 483.x 484.x 485 486	J12.xx J13 J14 J15.xx J16.x J17 J18.x
Non-infection-related cough	Cough	1440.0	786.2	R05
	Chronic URTD	-	472.x 473.x 476.0	J31.x J32.x J37.0

Abbreviations: URTI: upper respiratory tract infection; URTD: upper respiratory tract diseases

Pr Proprietary

Table S2. Multum Lexicon Plus® generic codes used to identify opioid antitussives, benzonatate, dextromethorphan-containing antitussives, gabapentinoids, and opioid analgesics in NAMCS/NHAMCS

Medication class and drugs	NAMCS	NHAMCS
Opioid antitussives[a]		
Codeine-containing antitussives	d03393 d03357 d00012 d03364 d03398 d03394 a11072 a11066 a11065 a11047	d03393 d03357 d00012 d03364 d03398 d03394 a11047 a11066 a11072
Dihydrocodeine-containing antitussives	a11555 d07525 d04904 d07127	a11555 a11557 a11713
Hydrocodone-containing antitussives	a10956 a10897 d03361 d03356 d03396 d03416 d03340 d03352 d03915 d03403 d04752 d03366 d03404 d03353 a11760 d04880 d05426 d05668	a10956 a10897 d03396 d03361 d03340 d03356 d03416 d03915 d03352 d03404 d03353 d03366 d03403 d04752 d04880 d05426
Benzonatate	d00796 a10928	d00796 a10928
Dextromethorphan-containing antitussives	d03400 d03368 a11259 d03360 d00207 d05366 d03409 d03370 d03408 d03372 d03577 d03411 d04165 d03371 d03908 d03359 d03354 d07707 a10032 a11269 d04818 d05150 a11224 d03355 a10501 a11261 a11271 a11273 a11274 d03342 d04339 d07297 a10497 a11196 a11265 d03344 d05814	d03400 d03368 d00207 d03360 d03577 d03409 a11259 d04165 d03370 d03411 d05366 d03349 d03372 d03408 a10032 d03354 d03908 a11273 d03359 a11266 a11269 d03371 d03378 a11223 d03344 d03355 d03940 d07707 a10444 a11221 a11224 a11261 a11270 a11271 d04339
Gabapentinoids		
Gabapentin	d03182	d03182
Pregabalin	d05508	d05508
Opioid analgesics[b]		
Codeine	d03423 d03425 d03426 d03424 a11076	d03423 d03425 d03426 d03424
Hydrocodone	d03428 d03075 d04225 a11768	d03428 d03075 d04225 a11768
Dihydrocodeine	d04269 d03168	d04269 d03168
Morphine	d00308	d00308
Oxycodone	d03431 d00329 d03432	d03431 d00329 d03432
Hydromorphone	d00255	d00255
Oxymorphone	d00833	d00833

Abbreviations: NAMCS: National Ambulatory Medical Care Survey; NHAMCS: National Hospital Ambulatory Medical Care Survey. [a] These medications are classified as '124: antitussives' or '132: upper respiratory combinations' by Multum Lexicon Plus®. [b] These medications are classified as '060: narcotic analgesics' or '191: narcotic analgesic combination' by Multum Lexicon Plus®.

Table S3. Patient, visit, and practice characteristics of visits hydrocodone- and codeine-containing antitussive use: 2003–2018 NAMCS (weighted-estimate of 819.9 million)

**Weighted visits**	**Hydrocodone-containing antitussives**	**Codeine-containing antitussives**	
	**30.4 million (3.7%)**	**33.0 million (4.0%)**	
**Characteristics**	**Weighted %**	**Weighted %**	**ASD[a]**
Age ≥65 years	23.2	23.7	0.07
Sex			0.03
Female	67.3	62.3	
Male	32.7	37.7	
Race			0.15
White	86.9	75.4	
Non-White	13.1	24.6	
Smoking status[b]			0.06
Current	15.1	15.8	
Non-current	81.6	81.0	
Payment source[c]			0.15
Governmental	25.4	29.7	
Commercial	66.7	58.8	
Others	6.3	8.8	
Chronicity of principal reason-for-visit[d]	13.6	12.1	0.04
≥ 2 Chronic conditions[e]	33.0	45.1	0.11
Top 3 major reasons-for-visit			
	Cough 69.5	Cough 61.4	
	Nasal congestion 24.6	Nasal congestion 19.4	
	Throat symptom 18.6	Throat symptom 18.4	
Top 3 major diagnoses			
	Bronchitis 42.3	Acute URTI 36.7	
	Acute URTI 37.1	Bronchitis 36.1	
	Chronic URTD 13.5	Cough 19.1	
Prescriber Specialty			0.04
Primary care	87.9	89.4	
Others	12.1	10.6	
Geographic region[f]			0.77
Northeast	9.6	15.2	
Midwest	15.8	23.0	
South	62.6	27.1	
West	12.0	34.7	
Metropolitan area	83.9	89.2	0.01

Abbreviations: NAMCS: National Ambulatory Medical Care Survey; ASD: absolute standardized difference; URTI: upper respiratory tract infections; URTD: upper respiratory tract diseases. [a] ASD > 0.1 was considered as having non-negligible differences. [b] Non-concurrent smoker includes never smoker, former smoker, and unknown. [c] Others include all other types of insurance, uninsured, and unknown. [e] The number of chronic conditions was available in NAMCS since 2005. [f] The region was unavailable in NAMCS in 2018. [b, c, d, e] The percentage of missingness for smoking status, payment source, chronicity of major reason-for-visit, ≥2 chronic conditions among cough-related visits involving hydrocodone- or codeine-containing antitussive were ≤3.3% from 2005 to 2018.

Table S4. Patient, visit, and practice characteristics of visits hydrocodone- and codeine-containing antitussive use: 2003–2018 NHAMCS (weighted-estimate of 155.5 million)

Weighted visits	Hydrocodone-containing antitussives	Codeine-containing antitussives	
	4.0 million (2.6%)	5.7 million (3.7%)	
**Characteristics**	**Weighted %**	**Weighted %**	**ASD[a]**
Age ≥65 years	13.3	8.8	0.10
Sex			0.15
Female	65.8	61.3	
Male	34.2	38.7	
Race			0.09
White	75.1	68.4	
Non-White	24.9	31.6	
Payment source[b]			0.11
Governmental	34.6	40.3	
Commercial	34.7	30.0	
Others	28.8	28.1	
≥ 2 Chronic conditions[c]	20.6	26.4	0.04
Top 3 major reasons-for-visit			
	Cough 62.6	Cough 69.4	
	Fever 15.9	Throat symptoms 20.0	
	Nasal congestion 15.4	Fever 16.1	
Top 3 major diagnoses			
	Bronchitis 45.7	Bronchitis 43.9	
	Acute URTI 28.7	Acute URTI 28.8	
	Cough 15.3	Cough 14.4	
Geographic region			0.64
Northeast	12.3	12.9	
Midwest	12.8	24.9	
South	66.1	41.5	
West	8.8	20.7	
Metropolitan area[d]	77.7	77.7	0.01

Abbreviations: NHAMCS: National Hospital Ambulatory Medical Care Survey; ASD: absolute standardized mean difference. [a] ASD > 0.1 was considered as having non-negligible differences. [b] The percentage of missingness for payment source among cough-related visits involving hydrocodone- or codeine-containing antitussive were ≤1.9% from 2003 to 2018. Others include all other types of insurance, uninsured, and unknown. [c] The number of chronic conditions was available since 2012. The percentage of missingness for the variable of ≥2 chronic conditions among cough-related visits involving hydrocodone- or codeine-containing antitussive were ≤1.4% from 2012 to 2018. [d] The metropolitan area was unavailable in NHAMCS in 2012.

Figure S1. Trends in hydrocodone- and codeine-containing antitussive use among cough-related visits in office-based ambulatory care setting: 2003–2018 NAMCS

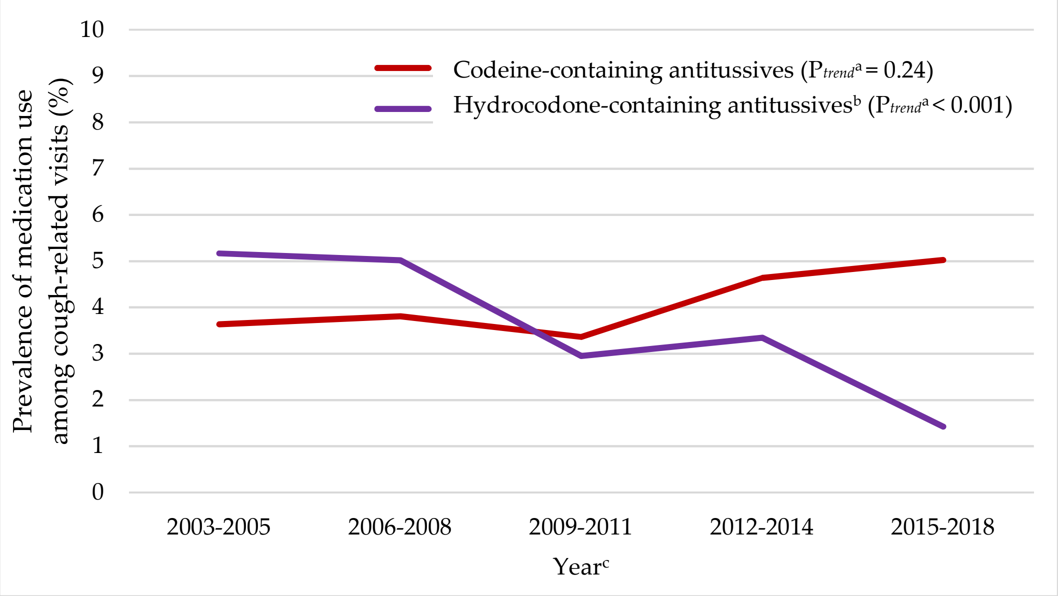

Abbreviations: NAMCS: National Ambulatory Medical Care Survey; NCHS: National Center for Health Statistics. [a] P_{trend} were adjusted for age, sex, race, and payment source. [b] The weighted-estimate for hydrocodone-containing antitussive in 2015–2018 is unreliable based on NCHS's recommendation. [c] The weighted-estimate of the denominator (i.e., adult cough-related visits) for each time period in NAMCS is 183.7 million, 171.9 million, 179.7 million, 152.8 million, and 131.8 million for 2003–2005, 2006–2008, 2009–2011, 2012–2014, and 2015–2018, respectively.

Figure S2. Trends in hydrocodone- and codeine-containing antitussive use among cough-related visits in ED setting: 2003–2018 NHAMCS

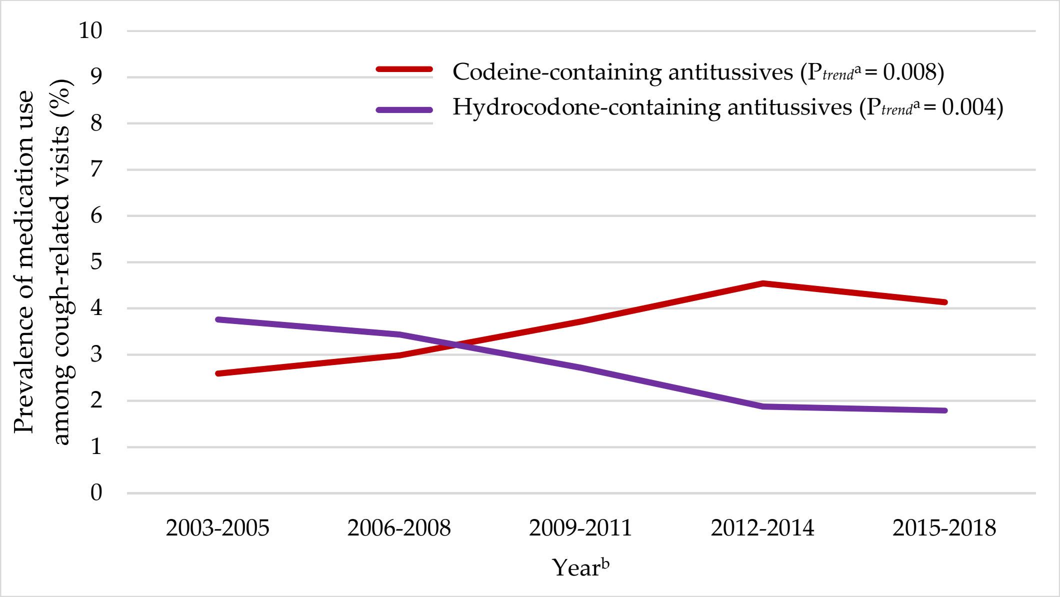

Abbreviations: NHAMCS: National Hospital Ambulatory Medical Care Survey; ED: Emergency Department. [a] P_{trend} were adjusted for age, sex, race, and payment source. [b] The weighted-estimate of the denominator (i.e., adult cough-related visits) for each time period in NHAMCS is 25.2 million, 25.8 million, 30.1 million, 30.7 million, and 43.7 million for 2003–2005, 2006–2008, 2009–2011, 2012–2014, and 2015–2018, respectively.

Pr Proprietary

Figure S3. Trends in prevalence of adult cough-specific visits among all adult visits in US ambulatory care settings: 2003–2018 NAMCS and 2003–2018 NHAMCS data

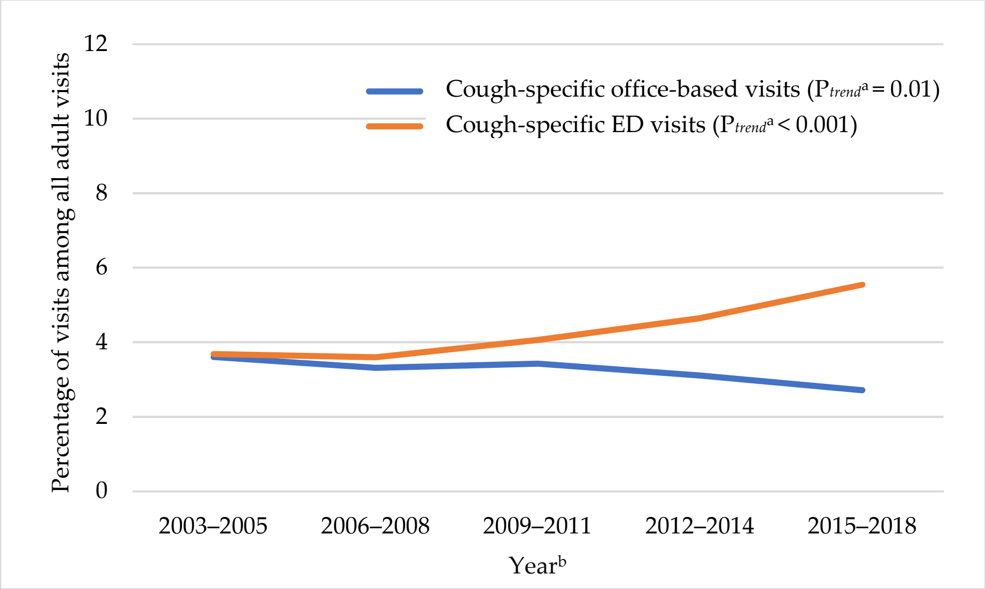

Abbreviations: NAMCS: National Ambulatory Medical Care Survey; NHAMCS: National Hospital Ambulatory Medical Care Survey; ED: Emergency Department. [a] P_{trend} were adjusted for age, sex, race, and payment source. [b] The weighted-estimate of the denominator (i.e., all adult visits) for each time period in NAMCS is 2.2 billion, 2.2 billion, 2.3 billion, 2.2 billion, and 2.2 billion for 2003–2005, 2006–2008, 2009–2011, 2012–2014, and 2015–2018, respectively. The weighted-estimate of the denominator (i.e., all adult visits) for each time period in NHAMCS is 252.9 million, 277.0 million, 308.1 million, 312.7 million, and 421.5 million for 2003–2005, 2006–2008, 2009–2011, 2012–2014, and 2015–2018, respectively.

Figure S4. Trends in medication use among cough-specific visits in office-based ambulatory care setting: 2003–2018 NAMCS

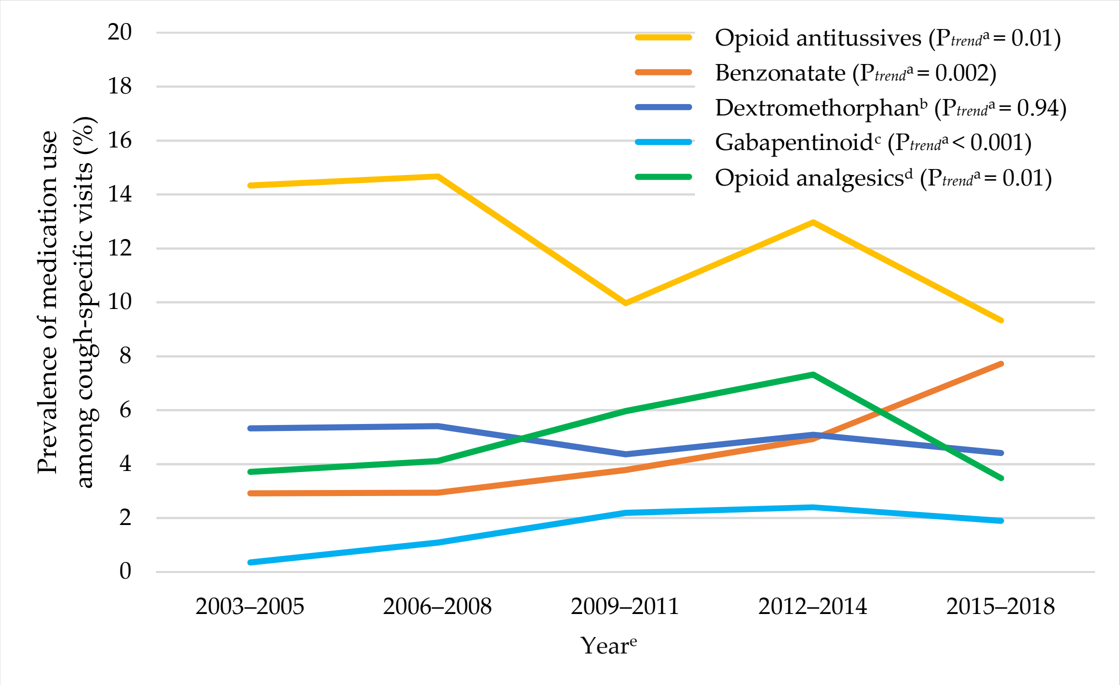

Abbreviations: NAMCS: National Ambulatory Medical Care Survey; NCHS: National Center for Health Statistics. [a] P_{trend} were adjusted for age, sex, race, and payment source. [b] Dextromethorphan indicates dextromethorphan-containing antitussives. The weighted-estimate for dextromethorphan in 2015–2018 is unreliable based on NCHS's recommendation. [c] The weighted-estimate for gabapentinoid in 2003–2005, 2006–2008, and 2015–2018 are unreliable based on NCHS's recommendation. [d] The weighted-estimate for opioid analgesics in 2015-2018 is unreliable based on NCHS's recommendation. [e] The weighted-estimate of the denominator (i.e., adult cough-specific visits) for each time period in NAMCS is 78.1 million, 72.6 million, 79.6 million, 68.6 million, and 59.7 million for 2003–2005, 2006–2008, 2009–2011, 2012–2014, and 2015–2018, respectively.

Figure S5. Trends in medication use among cough-specific visits in ED setting: 2003–2018 NHAMCS[a]

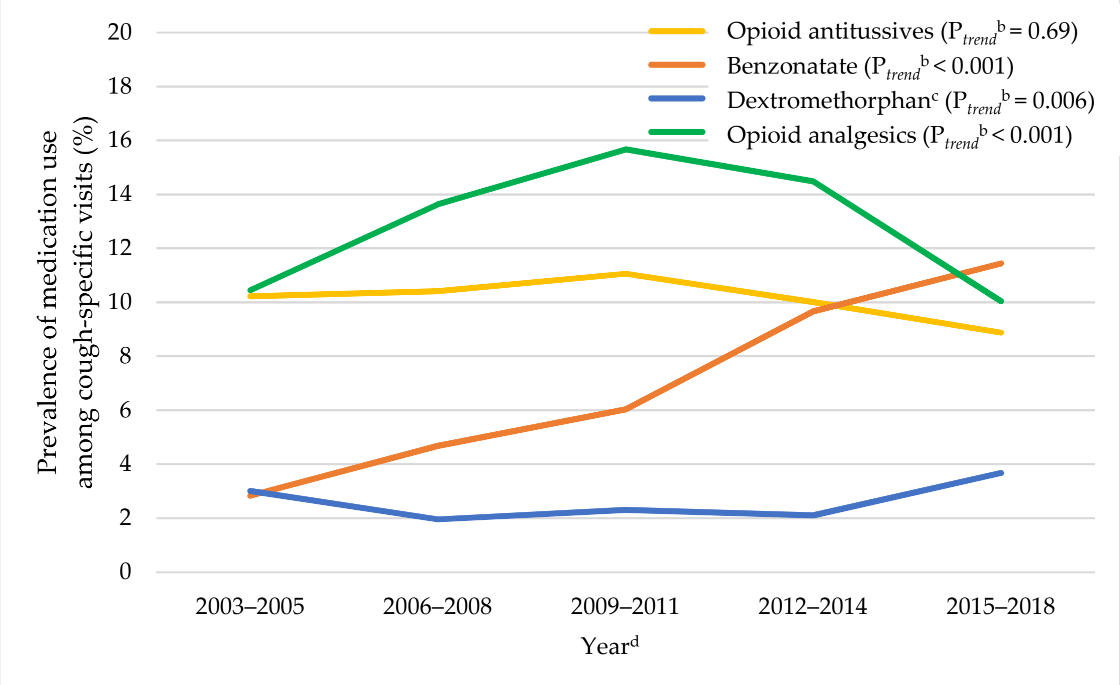

Abbreviations: NHAMCS: National Hospital Ambulatory Medical Care Survey; ED: Emergency Department; NCHS: National Center for Health Statistics. [a] The trend for gabapentinoid is not included; because, it had an unweighted count value of less than 30 in each time period, which is unreliable based on NCHS's recommendation. [b] P_{trend} were adjusted for age, sex, race, and payment source. [c] Dextromethorphan indicates dextromethorphan-containing antitussives. [d] The weighted-estimate of the denominator (i.e., adult cough-specific visits) for each time period in NHAMCS is 9.3 million, 10.0 million, 12.5 million, 14.5 million, and 23.4 million for 2003–2005, 2006–2008, 2009–2011, 2012–2014, and 2015–2018, respectively.

Pr Proprietary

Figure S6. Trends in medication use among all adult visits in office-based ambulatory care setting: 2003–2018 NAMCS

A. For all medications of interest

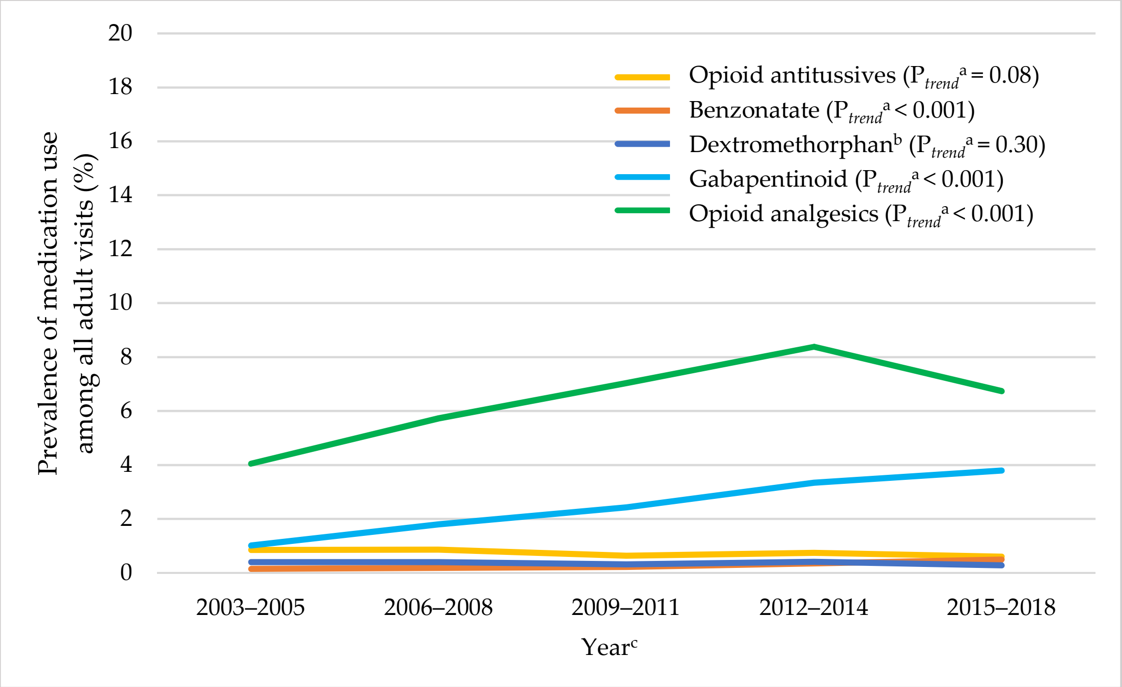

B. Enlarged figure for medications with prevalence less than 5%

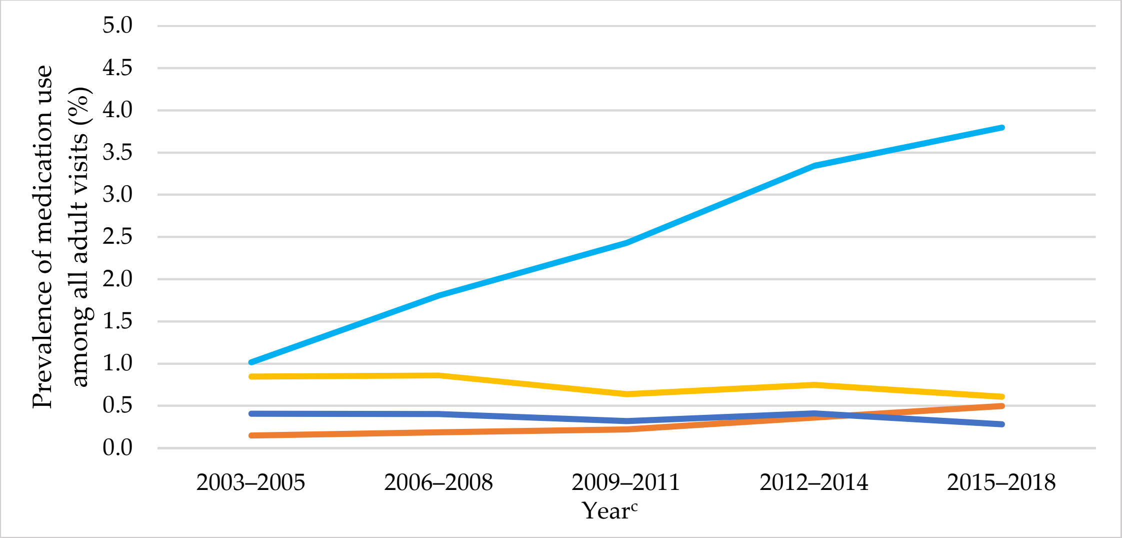

Abbreviations: NAMCS: National Ambulatory Medical Care Survey. [a] P_{trend} were adjusted for age, sex, race, and payment source. [b] Dextromethorphan indicates dextromethorphan-containing antitussives. [c] The weighted-estimate of the denominator (i.e., all adult visits) for each time period in NAMCS is 2.2 billion, 2.2 billion, 2.3 billion, 2.2 billion, and 2.2 billion for 2003–2005, 2006–2008, 2009–2011, 2012–2014, and 2015–2018, respectively.

Pr Proprietary

Figure S7. Trends in medication use among all adult visits in ED setting: 2003–2018 NHAMCS

A. For all medications of interest

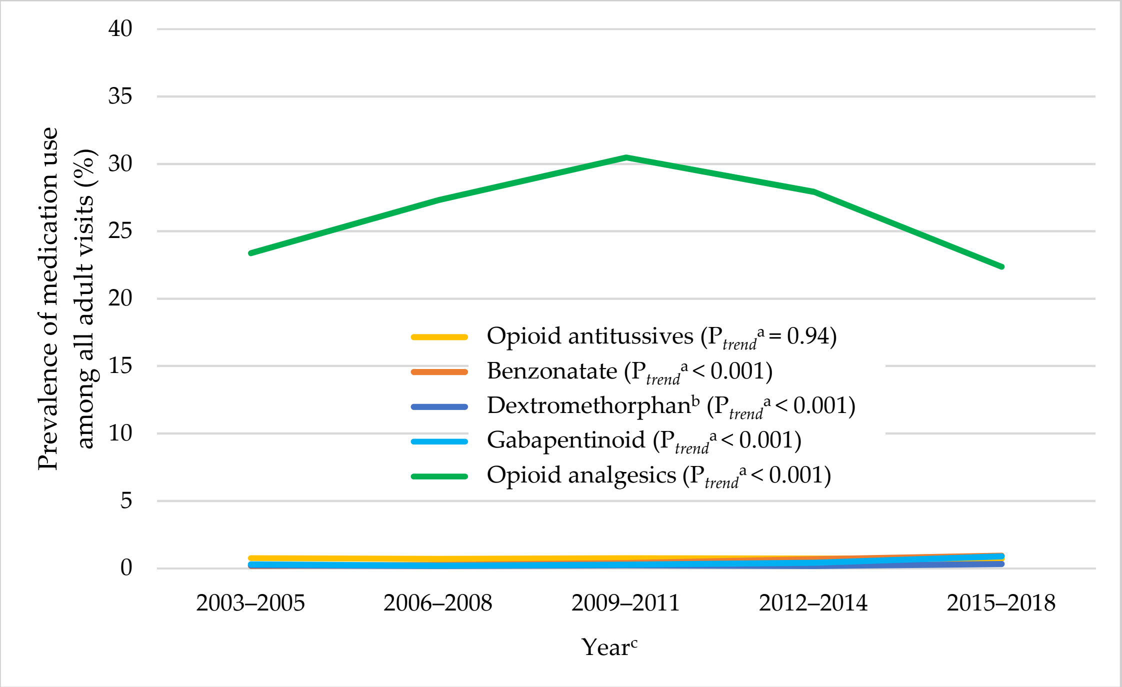

B. Enlarged figure for medications with prevalence less than 2%

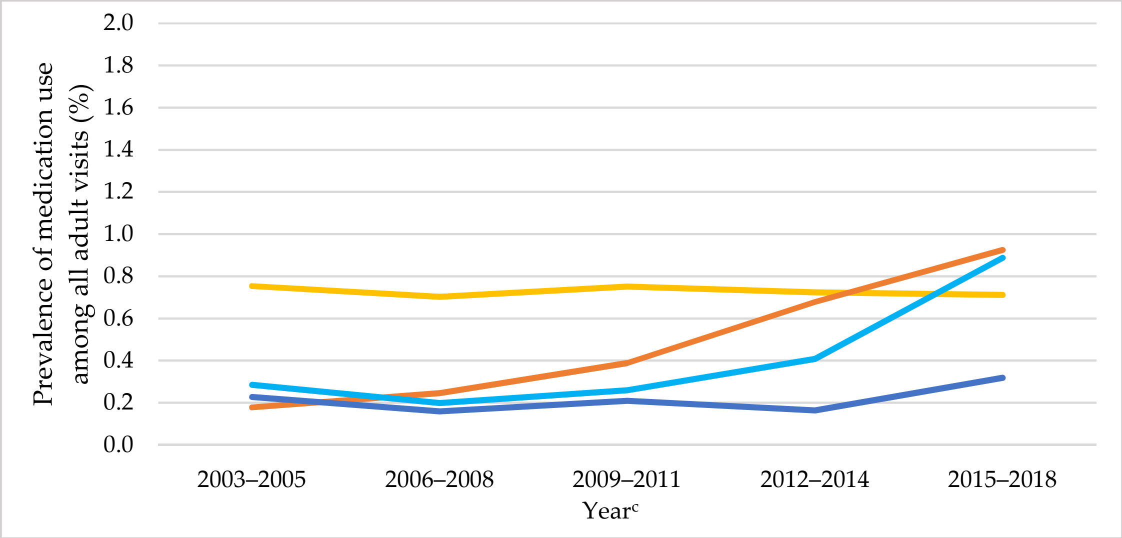

Abbreviations: NHAMCS: National Hospital Ambulatory Medical Care Survey; ED: Emergency Department. [a] P_{trend} were adjusted for age, sex, race, and payment source. [b] Dextromethorphan indicates dextromethorphan-containing antitussives. [c] The weighted-estimate of the denominator (i.e., all adult visits) for each time period in NHAMCS is 252.9 million, 277.0 million, 308.1 million, 312.7 million, and 421.5 million for 2003–2005, 2006–2008, 2009–2011, 2012–2014, and 2015–2018, respectively.
